# Impact of GnRH analogues on oocyte/embryo quality and embryo development in in vitro fertilization/intracytoplasmic sperm injection cycles: a case control study

**DOI:** 10.1186/1477-7827-7-103

**Published:** 2009-09-25

**Authors:** Ákos Murber, Péter Fancsovits, Nóra Ledó, Zsuzsa Tóthné Gilán, János Rigó, János Urbancsek

**Affiliations:** 1 1^st ^Department of Obstetrics and Gynaecology, Semmelweis University Faculty of Medicine, Budapest, Hungary

## Abstract

**Background:**

Despite the clinical outcomes of ovarian stimulation with either GnRH-agonist or GnRH-antagonist analogues for in vitro fertilization (IVF) being well analysed, the effect of analogues on oocyte/embryo quality and embryo development is still not known in detail. The aim of this case-control study was to compare the efficacy of a multiple-dose GnRH antagonist protocol with that of the GnRH agonist long protocol with a view to oocyte and embryo quality, embryo development and IVF treatment outcome.

**Methods:**

Between October 2001 and December 2008, 100 patients were stimulated with human menopausal gonadotrophin (HMG) and GnRH antagonist in their first treatment cycle for IVF or intracytoplasmic sperm injection (ICSI). One hundred combined GnRH agonist + HMG (long protocol) cycles were matched to the GnRH antagonist + HMG cycles by age, BMI, baseline FSH levels and by cause of infertility. We determined the number and quality of retrieved oocytes, the rate of early-cleavage embryos, the morphology and development of embryos, as well as clinical pregnancy rates. Statistical analysis was performed using Wilcoxon's matched pairs rank sum test and McNemar's chi-square test. P < 0.05 was considered statistically significant.

**Results:**

The rate of cytoplasmic abnormalities in retrieved oocytes was significantly higher with the use of GnRH antagonist than in GnRH agonist cycles (62.1% vs. 49.9%; P < 0.01). We observed lower rate of zygotes showing normal pronuclear morphology (49.3% vs. 58.0%; P < 0.01), and higher cell-number of preembryos on day 2 after fertilization (4.28 vs. 4.03; P < 0.01) with the use of GnRH antagonist analogues. The rate of mature oocytes, rate of presence of multinucleated blastomers, amount of fragmentation in embryos and rate of early-cleaved embryos was similar in the two groups. Clinical pregnancy rate per embryo transfer was lower in the antagonist group than in the agonist group (30.8% vs. 40.4%) although this difference did not reach statistical significance (P = 0.17).

**Conclusion:**

Antagonist seemed to influence favourably some parameters of early embryo development dynamics, while other morphological parameters seemed not to be altered according to GnRH analogue used for ovarian stimulation in IVF cycles.

## Background

The first IVF cycles were performed in natural unstimulated cycles [1]. Today gonadotrophins are administered to induce multiple follicular development and controlled ovarian hyperstimulation. During ovarian stimulation gonadotrophin-releasing hormone (GnRH) analogues are co-administered in order to prevent premature luteinizing hormone (LH) surges. Premature LH surges are observed in about 20% of stimulated cycles without using GnRH analogues [2,3]. Avoiding the adverse effects of elevated LH-levels, first GnRH agonist analogues were used to supplement the gonadotrophin stimulation. The continuous administration of GnRH agonists causes gonadotrophin suppression through down-regulation and desensitization of the GnRH receptors in the pituitary gland after an initial short period of gonadotrophin hypersecretion [4]. In 1985 the long protocol of GnRH agonists was reported [5]. Among other types of effective combined GnRH-agonist + gonadotrophin protocols, the long protocol proved to be the first choice of stimulation [6].

A decade later the first studies about the third, clinical adaptable generation of GnRH-antagonist analogues appeared [7,8]. GnRH antagonists (cetrorelix and ganirelix) cause immediate and rapid gonadotrophin suppression by competitive antagonism of the GnRH receptor in the pituitary without an initial period of gonadotrophin hypersecretion. Several advantageous effects (shorter stimulation period, lower risk of ovarian hyperstimulation syndrome) of cetrorelix were established [9], and these effects seemed to be independent from the type of antagonist used for LH-suppression [10]. Although combined GnRH antagonist and gonadotrophin stimulation represents an effective alternative to classical protocol with GnRH agonists, still the GnRH-agonist long protocol remained the first choice in the most IVF centres [11].

The quality of oocytes [12-15] and developing preembryos [16,17] is one of the most relevant factors determining the success of an IVF treatment. In order to improve the efficacy of the treatment, either more embryos at a time will be transferred or a well-established stimulation protocol and embryo-selection procedure with lower number of transferred embryos is practised. There is the need to transfer less but more viable embryos to reduce the occurrence of multiple pregnancies. As a result of improved fertilization and embryo culture techniques, patients may produce more good-quality embryos and have higher implantation and pregnancy rates. As ovarian stimulation protocol is one of the eligible factors during an IVF treatment, its embryo quality influencing effects are necessary to know. Since 2000 the comparison of GnRH agonist vs. GnRH antagonist protocols has been well analyzed in clinical studies [18-20], most of them focused on the clinical outcome of the two protocols, nevertheless the effects of the GnRH analogues on oocyte- and embryo-quality and on embryo development are still not known in detail.

The aim of our study was to verify the impact of the multiple dose protocol of GnRH antagonists in comparison with the long protocol of GnRH agonists on oocyte-, embryo quality and embryo development in IVF/ICSI cycles.

## Methods

### Subject groups

This retrospective case control study was performed with data of patients entering the IVF/ICSI program of the Division of Assisted Reproduction, First Department of Obstetrics and Gynecology, Semmelweis University School of Medicine, Budapest, Hungary. All IVF cycles performed between July 2001 and December 2008 were included in the antagonist-group (ANT) when GnRH antagonist was used during ovarian stimulation in the first IVF treatment cycle of the patient.

In selecting the control agonist-group (AG), it was determined to vary in only one clinical parameter from the antagonist group: the use of either GnRH agonist or GnRH antagonist during ovarian stimulation. All other clinical parameters (female age, body mass index [BMI], basal follicle-stimulating hormone [FSH] level, indication for IVF treatment, type of gonadotrophin used for ovarian stimulation) were matched to the antagonist pairs.

The study includes data from the first IVF/ICSI cycle of each patient only. Cancelled stimulations (no oocyte retrieval was performed) and IVF treatments completed with preimplantation genetic diagnosis (PGD) were excluded from the study.

All patients entering our department signed informed consent accepting their data will be used for scientific evaluations.

### Ovarian stimulation

In the antagonist group (ANT) multiple dose GnRH antagonist regimen (Lübeck protocol) was used for ovarian stimulation: 0.25 mg/day cetrorelix (Cetrotide; Serono, Rome, Italy) or ganirelix (Orgalutran; Organon, Oss, The Netherlands) was administrated from the fifth day of ovarian stimulation or from the presence of a follicle with 14 mm diameter. In the agonist group (AG) the long protocol of GnRH agonist was used: pituitary desensitization was achieved with GnRH agonist triptorelin (Decapeptyl; Ferring, Kiel, Germany), at a dose of 0.1 mg/day from the midluteal phase of the cycle preceding the treatment cycle.

Human menopausal gonadotrophin (HMG) (Menogon; Ferring or Menopur; IBSA, Lugano, Switzerland or Merional; IBSA) or high-purified urofollitropin (Fostimon HP; IBSA) was used for ovarian stimulation in both groups, which was monitored 1-2 times daily by estradiol measurements and transvaginal ultrasound examination. Type of gonadotrophin used for ovarian stimulation was always the same in one stimulation cycle; we have neither changed nor used gonadotrophins in line with an other type during one cycle.

Ovulation was induced with 5,000-10,000 IU of HCG (Profasi; Serono or Choragon; Ferring) when at least one follicle with a diameter of ≥18 mm and three or more follicles with a diameter of ≥16 mm were present and serum estradiol levels reached 2-300 pg/ml per ≥10 mm follicle. Transvaginal ultrasound-guided oocyte retrieval was performed 36 hours after HCG administration. 600 mg micronized progesterone (Utrogestan; Besins-Iscovesco Pharmaceuticals, Paris, France) intravaginally was used daily for luteal phase support.

### Sperm preparation and fertilization

Progressive motile sperms for insemination were isolated by swim up technique or by a two-layer density gradient centrifugation according to the quality of the native semen sample.

Conventional IVF was performed routinely 6 hours after oocyte retrieval (day 0). Motile sperm count used for insemination was 100-500.000 according to the patient age and semen quality. Following 16-18 hours coincubation oocytes were mechanically denuded of their cumulus cells and placed into culture media individually in separate wells of a four-well dish.

ICSI treatment was performed 5-8 hours after oocyte retrieval. The indications for ICSI were (A) <1 million progressive motile sperm after preparation and/or (B) ≤4 oocytes retrieved. Denudation of oocytes was performed by gentle pipetting after a short incubation in 80 IU/ml hyaluronidase. After sperm injection, the oocytes were placed into culture media individually.

Maturity of oocytes and cytoplasmic abnormality of oocytes were examined before sperm injection. Cytoplasmic alterations were rated abnormalities if oocytes contained large (≥10 μm) vacuoles or excessive granularity.

### Embryo culture

Embryos were cultured in home made Whittingham's T6 culture media [21] supplemented with 15% maternal serum or Vitrolife IVF culture media. Zygotes and embryos were cultured individually in 1 ml of culture media under 5% CO2 in air until embryo transfer was performed.

### Zygote and embryo assessment

16 to 20 hours after conventional IVF or ICSI (day 1) normal fertilization was confirmed by the presence of two pronuclei. At the same time pronuclear morphology were scored as it was described by Tesarik and Greco [22]: number and alignment of nucleoli (nucleolar precursor bodies, NPB) were evaluated and "Pattern 0" are called normal pronuclear morphology zygotes. Zygotes were examined again 22-25 hours after insemination or microinjection to determine whether pronuclear breakdown or first cleavage had occurred. On day 2 at 40-48 hours postinsemination embryos were assessed for cell number, uniformity of blastomere size, amount of fragmentation and incidence of the multinucleated blastomeres. The morphology score given to the embryos was: 4 for regular blastomeres, no fragments, and no multinucleated blastomeres; 3 for regular blastomeres, ≤20% fragments, and no multinucleated blastomeres; 2 for unequal-sized blastomeres or >20% fragments; 1 for unequal-sized blastomeres or >50% fragments; 0 for >80% fragmentation or no visible blastomeres. Embryos were termed top quality if their morphology score was 3 or 4 and they contained at least four cells on day 2.

### Embryo transfer and pregnancy

Our embryo transfer policy was the same in all of the cycles: embryos with the highest cell number and the highest grade were selected for embryo transfer. We transferred 1-3 embryos at once according to the age of the patient and embryo quality. Four embryos were transferred if the patient was more than 40 years of age. Supernumerous embryos with eligible morphology were cryopreserved.

Clinical pregnancy was considered according to WHO and ICMART (International Committee for the Monitoring of ART) definition of it as it is recommended by ESHRE: evidence of pregnancy by ultrasound visualisation of a gestational sac at gestational week 5-7.

### Statistical analysis

The statistical analysis was performed by Statistica 8.0 Software (StatSoft Inc., Tusla, USA). Mann-Whitney U-test or Wilcoxon's matched pairs rank sum test was used to compare mean values and Pearson chi-square or McNemar chi-square analysis for comparison of proportional values. *P *< 0.05 was considered statistically significant.

## Results

The two groups did not differ significantly with respect to baseline characteristics. Mean patient age, BMI, basal FSH concentration and cause of infertility was similar in the two groups (Table 1).

**Table 1:** Baseline characteristics of the patients

**Baseline parameters**	**GnRH antagonist**	**GnRH agonist**	***P *value**
**Female age (year)**	33.6 (± 5.6)	33.7 (± 5.6)	*0.74*
**BMI (kg/m^2^)**	23.0 (± 3.8)	22.9 (± 3.7)	*0.41*
**Basal FSH (IU/L)**	7.4 (± 4.1)	6.8 (± 1.8)	*0.07*
**Cause of infertility:**			
tubal factor	22	22	
other female origin	5	5	
male factor	35	35	
both female and male factor	14	14	
unknown origin	24	24	
**Total**	100	100	

BMI: body mass index; FSH: follicle stimulating hormone

The type of gonadotrophin used for ovarian stimulation was similar in the two groups. The clinical parameters of ovarian stimulation showed that significantly less ampoules of HMG were needed in the ANT group than in AG group and the length of stimulation was significantly longer in the AG group (Table 2).

**Table 2:** Characteristics of ovarian stimulation

**Ovarian stimulation**	**GnRH antagonist**	**GnRH agonist**	***P *value**
**Type of gonadotrophin used for stimulation**			
high purified urofollitropin	24	17	*0.29*
human menopausal gonadotrophin	76	83	
**Total**	100	100	
number of HMG ampoules (mean + SD)	25.9 (± 15.1)	31.5 (± 15.8)	*< 0.01*
Length of stimulation (days) (mean + SD)	8.9 (± 1.4)	10.5 (± 1.3)	*< 0.01*

HMG: human menopausal gonadotrophin

The number of follicles aspirated and oocytes retrieved was significantly lower in the antagonist group than with the use of the agonist. There was one cycle in the ANT group and four cycles in the AG group where we could not found oocytes at all. Rate of mature (MII) oocytes was similar in the two groups. We observed higher rate of cytoplasmic abnormalities in retrieved oocytes in the antagonist group than in agonist cycles (Table 3).

**Table 3:** Parameters of oocyte retrieval and of retrieved oocytes

**Oocyte characteristics**	**GnRH antagonist**	**GnRH agonist**	***P *value**
follicles aspirated (mean + SD)	9.0 (± 4.9)	11.2 (± 5.0)	*< 0.01*
cycles where no oocytes were found	1/100	4/100	*0.37*
retrieved oocytes (mean + SD)	6.5 (± 4.0)	8.1 (± 4.3)	*< 0.01*
mature (metaphase II.) oocytes (ICSI only) (%)	87.2 (340/390)	88.4 (419/474)	*0.58*
oocytes with cytoplasmic abnormalities (ICSI only) (%)	62.1 (208/335)	49.9 (228/457)	*< 0.01*

The method of fertilization was similar in both groups, ICSI was performed about three quarters of all cases. The percentage of normal fertilized oocytes (2PN zygotes) was similar in both groups independent of the method of fertilization. Having significant less oocytes available for fertilization in the antagonist group, the mean number of normal fertilized oocytes were also lower in this group. We observed higher rate of zygotes showing normal nucleolar distribution with the use of GnRH agonist analogues (Table 4).

**Table 4:** Rate of fertilization method, of normal fertilization and of nucleolar distribution

**Fertilization**	**GnRH antagonist**	**GnRH agonist**	***P *value**
ICSI frequency (%)	74.8 (74/99)	68.8 (66/96)	*0.22*
normal fertilization (all) (%)	55.9 (363/649)	60.4 (489/810)	*0.09*
normal fertilization (ICSI) (%)	57.7 (225/390)	59.9 (284/474)	*0.51*
normal fertilization (IVF) (%)	53.3 (138/259)	61.0 (205/336)	*0.06*
number of fertilized oocytes/cycle (mean + SD)	3.7 (± 3.0)	5.1 (± 3.4)	*< 0.01*
normal pronuclear morphology (%)	49.3 (176/357)	58.0 (280/483)	*< 0.01*

Using antagonist GnRH analogue for stimulation, we observed higher cell number of praeembryos on day 2 after fertilization, althought the higher rate of early-cleavage embryos did not reach statistical significance between the two groups. Contrary to the dynamics of embryo development, the percentage of top-quality embryos and the rate of presence of multinucleated blastomers this day seemed to be similar in the two groups, while the rate of praeembryos' fragmentation was lower using antagonists (Table 5).

**Table 5:** Characteristics of embryo development and embryo quality

**Embryo development**	**GnRH antagonist**	**GnRH agonist**	***P *value**
presence of early cleavage (%)	39.6 (108/273)	32.8 (139/424)	*0.07*
number of blastomers on day 2	4.28 (± 1.39)	4.03 (± 1.34)	*< 0.01*
top quality embryos on day 2 (%)	23.9 (84/351)	26.8 (128/477)	*0.34*
presence of multinucleated blastomers (%)	13.7 (49/357)	13.0 (62/477)	*0.76*
amount of fragmentation on day 2 (%)	15.2 (± 10.2)	17.3 (± 12.0)	*< 0.01*

Clinical pregnancy rates were in tendency lower in the antagonist group than in the agonist group (per embryo transfer *P *= 0.17 and per stimulation cycles *P *= 0.13), implantation rates were also lower using antagonists although these differences did not reach statistical significance. The odds ratios for clinical pregnancy rate were per transfer 0.65 (0.34-1.25) and per cycle 0.63 (0.33-1.20).

The mean number of supernumerous embryos appropriate for cryopreservation seemed to be similar in the two groups (Table 6).

**Table 6:** Clinical outcomes in the GnRH antagonist and GnRH agonist groups

**Clinical outcome**	**GnRH antagonist**	**GnRH agonist**	***P *value**
embryotransfer performed (%)	91.0 (91/100)	94.0 (94/100)	*0.58*
transferred embryos (mean + SD)	2.59 (± 0.87)	2.84 (± 0.83)	*0.06*
cryopreservation performed (%)	17.0 (17/100)	28.0 (28/100)	*0.09*
cryopreserved embryos (mean + SD)	4.29 (± 1.92)	4.64 (± 2.04)	*0.57*
clinical pregnancy rate (%)/ET	30.8 (28/91)	40.4 (38/94)	*0.17*
clinical pregnancy rate (%)/cycle	28.0 (28/100)	38.0 (38/100)	*0.13*
implatation rate (%)	19.1 (45/236)	20.6 (55/267)	*0.67*

## Discussion

The success of an IVF/ICSI treatment depends substantially on the quality of transferred embryos. Among numerous factors affecting embryo quality, ovarian stimulation is an eligible and adjustable one. Despite the established clinical impact of different stimulation protocols, analysis of ovarian stimulation on quality of oocytes and developing embryos is not well known yet.

Nevertheless since before 2000 the combination of GnRH antagonists and gonadotrophins has also been available, the GnRH-agonist long protocol remained the first choice in most IVF centres [11] as it is in our IVF department, too. We choose GnRH antagonist protocols at the first IVF cycle of the patient almost exclusively when the duration of the pretreatment and ovarian stimulation is limited; this explains the small number of patients in our study in spite of the relatively long trial period.

As previous studies reported [19,20], we also showed that patients need less HMG ampoulles and the length of stimulation is shorter with the use of GnRH antagonists protocol. We aspirated significantly more follicles and we retrieved significantly more oocytes with the use of GnRH agonist; most of the comparative studies of GnRH analogues had similar results [9,19,20].

These clinical aspects have been evaluated in several studies, but only a recent study focused on the differences in embryo quality according to the type of gonadotrophin used for ovarian stimulation [23].

Like in some previous studies, there was no significant difference between the rate of mature metaphase II oocytes in the two groups in our study, however this parameter was examined only by a few workgroups and one study was made on a special group of non-obese PCOS patients [18,19,24].

Several studies focused on the role of oocyte quality in predicting treatment outcome. Granularity in the perivitelline space seems to be a physiological phenomenon in oocytes and it could be enhanced by exposure to high dosages of gonadotrophins [25]. In a recent study stimulation protocol prooved to influence significantly the zona pellucida score (agonist protocol resulted in better score compared to the antagonist one) [26]. The presence of intracytoplasmic abnormalities can refer to the quality of the oocyte [12,13]. Otsuki et al. confirmed pronucleus sized translucent vacuoles in oocytes as tubular-type smooth endoplasmic reticulum clusters (sERCs) [14]. sERC positive oocytes were observed more frequent in GnRH agonist short protocols compared to long ones. Comparing GnRH agonist long proctocol to GnRH antagonist cycles in our study we have also found significantly less oocytes with cytoplasmic abnormalities after administrating GnRH agonists in long protocol.

According to the method of fertilization (IVF or ICSI) there was no significant difference between the two groups in the rate of normally fertilized oocytes; this parameter was similar in both groups independent of the method of fertilization. In previous studies the rate of the normally fertilized oocytes was examined during conventional IVF and ICSI treatments together only: cumulatively there was no significant difference between the two groups [20]. However these parameters were similar in the two groups, accordingly the lower count of retrieved oocytes, the average number of normally fertilized zygotes was significantly lower in the antagonist cycles. Normal nucleolar distribution was also significantly lower in the antagonist group. This parameter has not been examined yet in previous studies in view to the type of GnRH analogue used for stimulation. The zygotes with normal pronuclear morphology are supposed to develop most likely top-quality embryos [27-29].

Dynamics of early embryonic development could reflect the developmental potential of the embryo. The first cleavage can be examined with the breakdown of the pronuclear membrane (the start of the M phase of the first cell cycle) and directly with the presence of cleavage, because the duration of the M phase is relatively constant (3-4 hours) [30,31]. It is known that early cleavage is a strong indicator of the quality and the viability of the embryos [30,32,33], although a recent study showed higher implantational potential for early-cleavage embryos only with the use of GnRH agonists [34]. We observed significantly higher number of blastomers in the antagonist group on day 2, while the higher presence of early cleavage in this group did not reach statistical significance.

There was no significant difference in the rate of the top quality embryos. (Embryo quality and development were examined also on day 3 [64-72 hours postinsemination], but only the results of day 2 are analyzed in this study, because part of the embryos are used to be transferred on day 2 already. Hence the results of day 3 would not be representative in this study). Other studies had similar results in the rate of the top quality embryos, but the early cleavage, the number of multinucleated blastomeres (multinucleated embryos have poor implantation potential [35]) and the amount of fragmentation have not been yet examined during comparative studies of GnRH analogues.

The clinical pregnancy rate, which shows the effectiveness of the treatments, was examined in all of the comparative studies. Co-administrating GnRH agonists during gonadotrophin ovarian stimulation seems to result in higher pregnancy rates, however this difference proved not to be significant in most of the studies [19,20,24]. Despite of the not significant difference in our study in pregnancy rates, the presented odds ratios indicate higher pregnancy rates by GnRH agonists. The higher number of cycles with supernumerous embryos appropriate for cryopreservation in the agonist group favours the patient avoiding a repeated ovarian stimulation and oocyte retrieval procedure in a contingent next treatment.

## Conclusion

It seems that more oocytes can be retrieved, there is less cytoplasmic abnormality in the mature oocytes, there are more oocytes with normal fertilization and there are more zygotes with normal pronuclear morphology after stimulation with GnRH agonist analogues. In contrast, there are more blastomeres in the embryos on day 2 when GnRH antagonists were administered. While there was no significant difference between clinical pregnancy rates of the two groups, the advantageous and the disadvantageous effects of the GnRH analogues on the quality of the oocytes and the embryos may be equalized.

We hope the new advantages and disadvantages of the GnRH analogues identified through this study can be the principle starting further investigations to help the clinician choose the appropriate medication for ovarian stimulation in IVF/ICSI treatments.

## Competing interests

The authors declare that they have no competing interests.

## Authors' contributions

AM participated in the design of the study, collected the data and coordinated the study. PF participated in data collection, data interpretation and statistical analysis. NL helped in data collection and to draft the manuscript. ZGT carried out the laboratory analysis of oocytes and embryos. JU participated in the design of the study and in the critical revision of the manuscript. JR reviewed the manuscript. All authors read and approved the final manuscript.
